# Supplementary material for: Functional Characterization of Serum Amyloid P Component (SAP) in Host Defense against Bacterial Infection in a Primary Vertebrate
Source: Int J Mol Sci. 2022 Aug 22;23(16):9468. doi: 10.3390/ijms23169468 (PMC9409150; doi:10.3390/ijms23169468)
Supplement: Supplementary file 1 [file ijms-23-09468-s001.zip › ijms-1851847-supplementary.pdf]

Supplementary Table S1. The primers used in this study.

Primers	Sequence (5′-3′)	Purpose
OnSAP-F	GCACTCTTCCAACAATCT	Sequencing
OnSAP-R	CACTCTTCCAACAATCT	Sequencing
EOnSAP-F	CCGGAATTCAATAAAATATTCATCTTCCCACAACAA	Protein expression
EOnSAP-R	CCGAAGCTTCACTCTTCCAACAATCTGAAACTCA	Protein expression
qOnSAP-F	TCTCTGGCTACACCCTCTGCTAT	RT-qPCR
qOnSAP-R	ATCCCTTCAACTTTATTCGCCTTGC	RT-qPCR
T7	TAATACGACTCACTATAGGG	Sequencing
T7t	GCTAGTTATTGCTCAGCGG	Sequencing
β-actin-F	CGAGAGGGAAATCGTGCGTGACA	Control, RT-qPCR
β-actin-R	AGGAAGGAAGGCTGGAAGAGGGC	Control, RT-qPCR

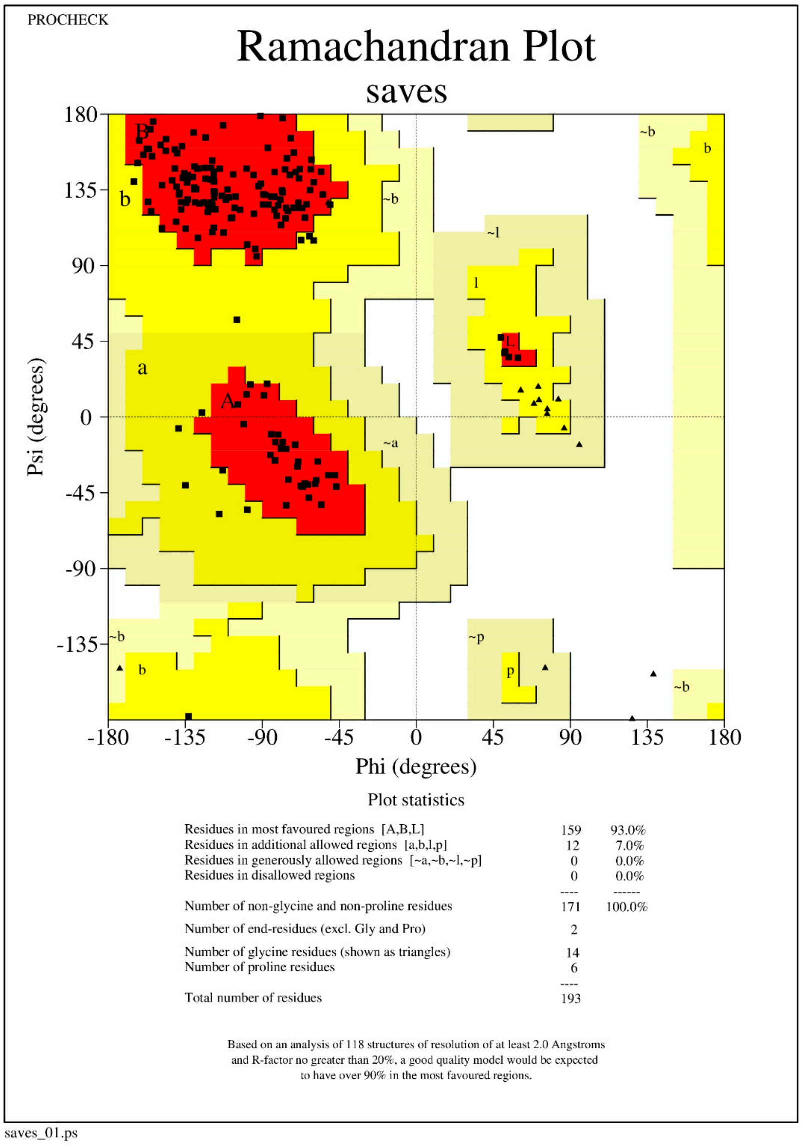

Supplementary Figure S1. Ramachandran PLOT analysis for OnSAP structure verification.
